# Is the association between blood pressure and mortality in older adults different with frailty? A systematic review and meta-analysis

**DOI:** 10.1093/ageing/afz072

**Published:** 2019-09-01

**Authors:** Oliver M Todd, Chris Wilkinson, Matthew Hale, Nee Ling Wong, Marlous Hall, James P Sheppard, Richard J McManus, Kenneth Rockwood, John Young, Chris P Gale, Andrew Clegg

**Affiliations:** 1 Academic Unit of Elderly Care and Rehabilitation, University of Leeds, Bradford Teaching Hospitals NHS Foundation Trust, Bradford BD9 6RJ, UK; 2 Clinical and Population Sciences Department, Leeds Institute of Cardiovascular and Metabolic Medicine, University of Leeds, UK; 3 Nuffield Department of Primary Care Health Sciences, University of Oxford, UK; 4 Geriatric Medicine, Dalhousie University, Canada

**Keywords:** hypertension, antihypertensive, older people, ageing, frail, systematic review

## Abstract

**Objective:**

to investigate whether the association between blood pressure and clinical outcomes is different in older adults with and without frailty, using observational studies.

**Methods:**

MEDLINE, EMBASE and CINAHL were searched from 1st January 2000 to 13th June 2018. PROSPERO CRD42017081635. We included all observational studies reporting clinical outcomes in older adults with an average age over 65 years living in the community with and without treatment that measured blood pressure and frailty using validated methods. Two independent reviewers evaluated study quality and risk of bias using the ROBANS tool. We used generic inverse variance modelling to pool risks of all-cause mortality adjusted for age and sex.

**Results:**

nine observational studies involving 21,906 older adults were included, comparing all-cause mortality over a mean of six years. Fixed effects meta-analysis of six studies demonstrated that in people with frailty, there was no mortality difference associated with systolic blood pressure <140 mm Hg compared to systolic blood pressure >140 mm Hg (HR 1.02, 95% CI 0.90 to 1.16). In the absence of frailty, systolic blood pressure <140 mm Hg was associated with lower risk of death compared to systolic blood pressure >140 mm Hg (HR 0.86, 95% CI 0.77 to 0.96).

**Conclusions:**

evidence from observational studies demonstrates no mortality difference for older people with frailty whose systolic blood pressure is <140 mm Hg, compared to those with a systolic blood pressure >140 mm Hg. Current evidence fails to capture the complexities of blood pressure measurement, and the association with non-fatal outcomes.

## Introduction

Improvements in cardiovascular care and global demographic changes mean that people are now more typically living into later life with hypertension. By the age of 80, more than three quarters of adults will have been diagnosed with hypertension [1]. However, life course trajectories of systolic blood pressure demonstrate deceleration and eventual decline in later life [2]. The association of blood pressure and the proportional risk of vascular mortality reduces with age [3]. Furthermore, anti-hypertensive treatment in older people can be associated with harm: higher rates of electrolyte disturbance, acute kidney injury [4], orthostatic hypotension, syncope and falls [5]. Evidence also indicates accelerated cognitive decline in patients with established mild cognitive impairment or dementia [6].

Little guidance is available to help practitioners identify patients for whom a less intensive approach to blood pressure management is appropriate. There are good reasons to consider a person’s frailty status when treating blood pressure. Frailty is better than chronological age in predicting all-cause mortality [7], primary cardiac end-points [8], and functional outcomes including disability, falls, and nursing home admissions [9]. People living with frailty can be especially prone to the adverse effects of medications [10].

There is an absence of the necessary randomised control trial (RCT) evidence on which to base clinical guidelines for this patient group. Therefore, a summary of observational studies is necessary, albeit with the caveat that their interpretation must account for their higher risk of reverse causality and residual confounding. Criteria for treatment and thresholds for diagnosis of hypertension vary across countries and over time. We therefore conducted a systematic review and meta-analysis of observational studies including older people living with frailty in the community, with and without antihypertensive treatment, to investigate whether the observed relationship between blood pressure and relevant clinical outcomes is different in the context of frailty.

## Methods

The review methodology followed the MOOSE guidance and is reported using Preferred Reporting Items for Systematic Reviews and Meta-Analyses (PRISMA) recommendations [11, 12] (Appendix 1). The protocol was prospectively registered with Prospero http://www.crd.york.ac.uk/PROSPERO/ (reference CRD42017081635).

### Inclusion criteria

Observational studies involving community-living older adults (mean age >65) and participant follow-up for at least 6 months. Blood pressure was measured at baseline with or without treatment, using a measurement standardised within the study. Frailty was defined as a state of vulnerability to adverse outcomes [7] and measured at baseline using a measure validated as being prognostic for reduced survival in more than one study. If a participant was unable to complete the frailty test, their data were excluded from meta-analysis. This is because non-participation does not represent a validated measure of frailty—non-completion of the test may be for reasons other than frailty.

The primary outcome was all-cause mortality. Planned secondary outcomes included: falls; stroke; non-fatal myocardial infarction; secondary prevention outcomes (e.g. proteinuria); adverse treatment effects; non-cardiovascular mortality; and other markers of general morbidity (including unplanned hospitalisation; institutionalisation; function; and quality of life).

### Search methods for identification of studies

An inclusive MEDLINE search strategy was developed with an experienced research librarian at the University of Leeds, and adapted for CINAHL, EMBASE, and Web of Science. All databases were searched for English language publications between 1st January 2000 and 13th June 2018. The search strategy for MEDLINE (Ovid SP) is available (Appendix 2). Reference lists of included articles were also searched. PROSPERO, Research registry and NIHR (National Institute of Health Research) research registries were searched for unpublished work. Authors were contacted if abstracts referred to unpublished work, and we completed a forward citation search of all included studies.

Study eligibility was determined by two independent reviewers (OT, and CW, or MP, or MH) with any disagreements settled by consensus discussion with a third reviewer (AC). Reasons for exclusion of articles at the full-text review stage were collated using Covidence software.

### Data extraction

We extracted hazard risks (HRs) with 95% confidence intervals (CIs) for time to event data (e.g. mortality) for different categories of baseline systolic and diastolic blood pressure, with and without frailty, adjusted for a minimum of age and sex. This process was performed independently by two reviewers (OT, CW). Any disagreements were settled by consensus discussion with a third reviewer (AC).

### Assessment of risk of bias

Two independent reviewers (OT, and CW or MH) assessed risk of bias for each study using the RoBANS tool [13].

### Meta-analyses

We synthesised data for meta-analysis by calculating natural logarithms of HRs, with standard errors to create summary forest plots by generic inverse variance random effects modelling using RevMan 5.3 software. We assessed statistical heterogeneity using the I^2^ statistic to determine whether fixed effects (*I*^2^ < 50%) or random effects (*I*^2^ ≥ 50%) modelling should be used. Since fewer than 10 studies were identified to provide data for each outcome, assessment for publication bias with funnel plots was not appropriate [14].

Where studies used different reference categories for blood pressure (BP), we re-categorised estimates comparing groups according to thresholds for treatment recommended by National Institute for Health and Care Excellence (NICE) guidelines (systolic BP of 140 mm Hg, and diastolic BP of 90 mm Hg) [15]. Where there was more than one category on either side of the threshold, risk estimates from directly neighbouring categories were extracted and pooled using generic inverse variance methods (Appendix 3). Data from ‘less than’ categories (<) were pooled with data from ‘less than or equal to’ (≤) categories and the same was done for ‘more than’ and ‘more than or equal to’ categories. Where continuous scales of measurement were used, the HR for events associated with 10 mm Hg difference in blood pressure at baseline was extracted.

## Results

### Literature search

**Figure 1:**
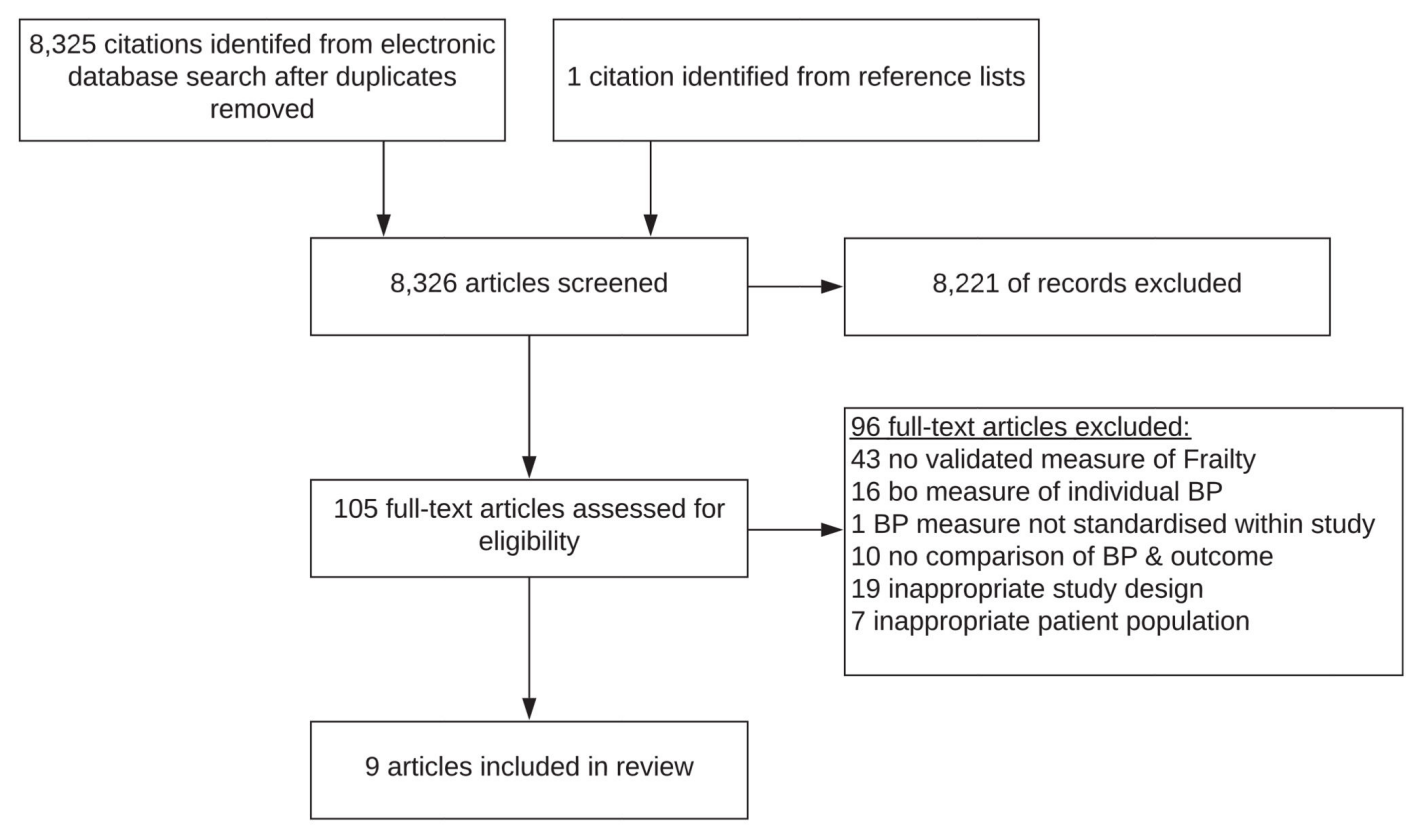
Flowchart of included studies.

Details of the study selection are presented in Figure 1. Following detailed assessment, nine studies were eligible for inclusion in the review; eight were included in the meta-analysis [16–23] of which seven required further information, which was supplied by study authors [16, 18–23]. It was not possible to make contact with the author of one study, which therefore had to be excluded from the meta-analysis [24]. A forward citation search on 8^th^ March 2019 revealed 91 studies, none of which met eligibility criteria.

### Study characteristics

The nine studies were all prospective cohort studies with a total of 21,906 participants and mean follow-up period of 6 years (range 3 to 11 years) (Table 1). All studies were rated as low or moderate risk of bias (Table 2). Three studies were based on study populations in the United States [18, 19, 23], five in Europe [16, 17, 20–22] and one in China [24] with study periods between 1989 and 2014. The studies recruited a mean of 58% (range 20–92%) of eligible participants. The mean age was 81 years (74–92 years) and 59% (51–70%) were female. In the four studies in which it was reported, care home residents constituted 24% (10–39%) of the study population [20–22], in two studies care home residents were excluded [18, 23]. Frailty was identified in 37% (13–64%) of participants, and antihypertensive use was reported in 52% (26–81%), and where reported, a diagnosis of hypertension in 48% (25–70%) [21, 24]. Median annual mortality for the whole study population was reported to be 7% (range 4–17%).

Each study compared both systolic and diastolic blood pressures, as an average of between one and four readings, all at the start of the study. Five studies analysed blood pressure as a continuous variable [18–21, 23] and seven studies categorised blood pressure [16–19, 22–24] into 2–5 groups using thresholds used in Joint National Committee (JNC) 7 [18], JNC 8 [23], or European Society for Cardiology (ESC) 2013 guidelines [16]. In studies that did not report blood pressure categories using thresholds according to NICE guidelines [15] we contacted study authors. Frailty was measured using a variety of measures, and categorised using different thresholds (See Table 1).

All nine studies reported all-cause mortality as an outcome, in eight as a primary outcome. One study reported cardiovascular morbidity as a primary outcome, and mortality as secondary outcome [19]. Other secondary outcomes included disease-specific mortality [24], cardiovascular mortality [21], and change in cognitive function [20].

Group consensus opinion was that study eligibility criteria and included populations were sufficiently similar to allow pooling of findings from eight studies (*n* = 17,248, mean duration 6 years) for comparison of all-cause mortality risk [16–23]. There were too few studies to allow meta-regression [25]. One study was excluded from meta-analysis because risk estimates were not reported for sub-groups with and without frailty [24].

### Risk of bias

Comprehensive assessment of the risk of bias using the RoBANS tool highlighted deficiencies, but overall risk of bias was low or moderate for each of the included studies (Table 2). Three studies gave incomplete information on cohort recruitment [17, 21, 23]. Four studies were rated as at high or unclear risk of bias for the measurement of exposure. In these, the frail sub-cohort included participants who were unable to complete the frailty test [16, 19, 20, 22]. In two studies the clinical or statistical justification for the choice of confounding variables was not reported [23, 24]. None of the studies referenced a published protocol with pre-specified methods. In all studies, mortality was determined by robust means – either death registries or primary care physician. Missing data for covariates was not accounted for with one exception [24]. In one study, more than 20% participants had some missing data on relevant covariates [16].

### Primary outcome—all-cause mortality

#### Categorical blood pressure comparisons

##### Systolic Blood Pressure

**Figure 2:**
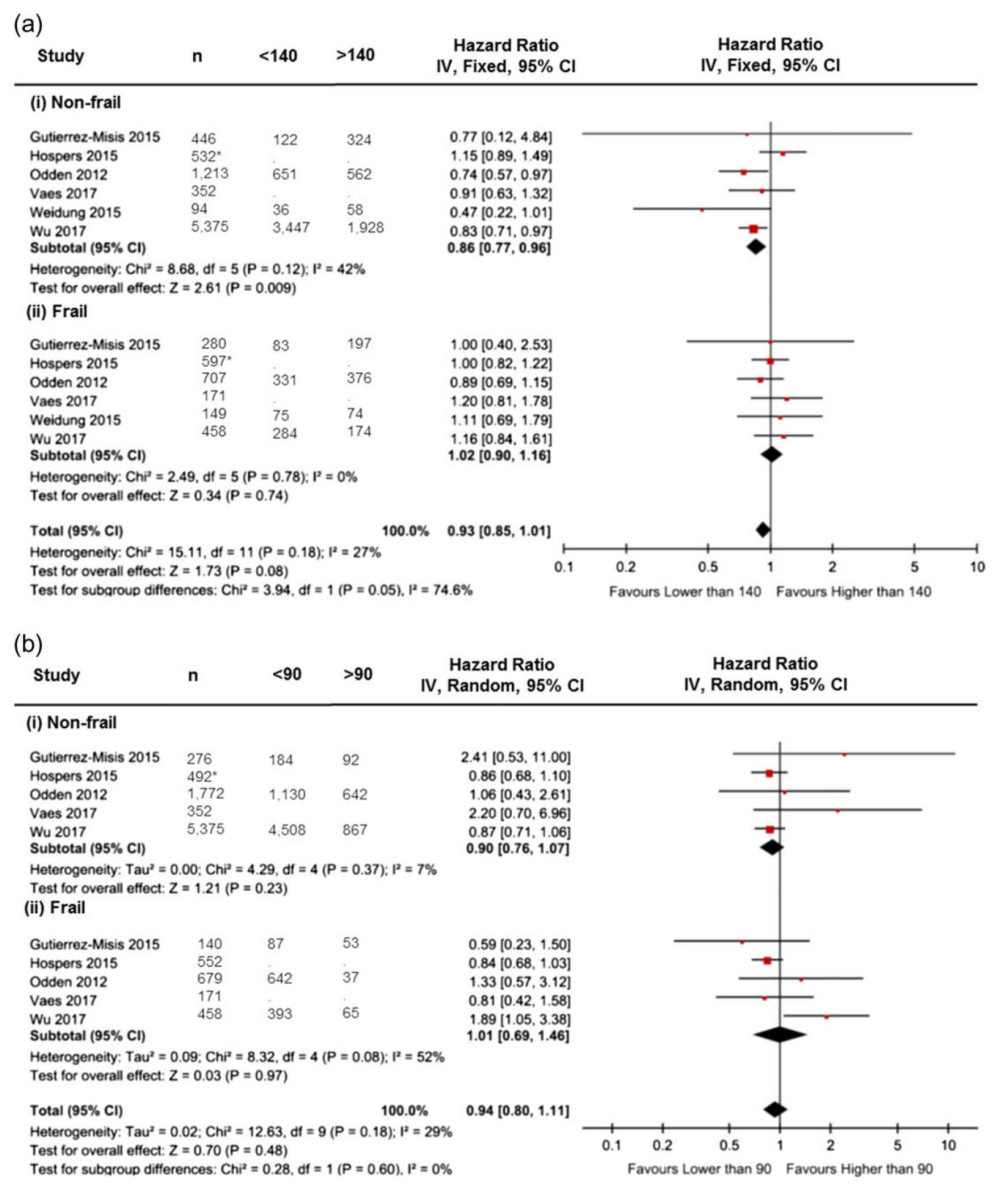
Forest plots demonstrate pooled risk estimates of all-cause mortality in older people with and without frailty (a) Association between all-cause mortality and systolic blood pressure <140 mm Hg compared to >140 mm Hg in older people without frailty (i) and older people with frailty (ii). (b) Association between all-cause mortality and diastolic BP < 90 mm Hg compared to >90 mm Hg in older people without frailty (i) and older people with frailty (ii). <90 = diastolic BP < 90 mm Hg; >90 = diastolic BP > 90 mm Hg; <140 = systolic BP < 140 mm Hg; >140 = systolic BP > 140 mm Hg; CI = Confidence Interval; Fixed = Fixed Effects; IV = Inverse Variance; n = study population size; Random = Random Effects; * = these numbers are estimated using aggregate numbers reported.

Synthesis of data from six cohort studies [16–18, 21–23] demonstrated that a systolic blood pressure less than 140 mm Hg had no association with mortality in older people with frailty compared to a systolic blood pressure more than 140 mm Hg (HR 1.02, 95% CI 0.90 to 1.16, *n* = 2,362) (Figure 2). However, in the absence of frailty, a systolic blood pressure lower than 140 mm Hg was associated with lower mortality compared to a systolic blood pressure of more than 140 mm Hg (HR 0.86, 95% CI 0.77 to 0.96, *n* = 8,012). There was no evidence of statistical heterogeneity across studies for sub-groups with frailty (*I*^2^=0%), and low heterogeneity in sub-groups without frailty (*I*^2^=42%).

##### Diastolic blood pressure

Synthesis of data from five cohort studies [16–18, 21, 23] demonstrated that a diastolic blood pressure lower than 90 mm Hg was not associated with a difference in mortality compared with a diastolic blood pressure greater than 90 mm Hg for those with frailty (HR 1.01, 95% CI 0.69 to 1.46, *n* = 2,000) or in those without (HR 0.90 95% CI 0.76 to 1.07, *n* = 8,267) (Figure 2). There was evidence of moderate heterogeneity for the sub-group with frailty (*I*^2^=52%), but not the sub-group without frailty (*I*^2^=7%) so a random effects meta-analysis was performed.

#### Continuous blood pressure comparisons

Pooled risk estimates were calculated for a 10 mm Hg difference in systolic blood pressure (from five studies, *n* = 12,280) [18–21, 23] and diastolic blood pressure (four studies, *n* = 11,668) [18, 19, 21, 23].

##### Systolic blood pressure

A 10 mm Hg difference in systolic blood pressure had no association with mortality in people with frailty (HR 1.02, 95% CI 0.96 to 1.07, *n* = 3,138) orthose without frailty (HR 1.00, 95% CI 0.97 to 1.04, *n* = 9,142). There was evidence of heterogeneity in the association of continuous measurements of systolic blood pressure and mortality for both the sub-groups with frailty (*I*^2^=68%), and without frailty (*I*^2^=27%) so a random effects meta-analysis was performed.

##### Diastolic blood pressure

Similarly, a 10 mm Hg difference in diastolic blood pressure was not associated with mortality in people with frailty (HR 1.02, 95% CI 0.97 to 1.07, *n* = 2,748) or without frailty (HR 0.95, 95% CI 0.91 to 1.00, *n* = 8,920). There was no evidence of heterogeneity in the association of continuous measurements of diastolic blood pressure and mortality for both the sub-groups with frailty (*I*^2^=0%), and without frailty (*I*^2^=0%) so a fixed effects meta-analysis was performed.

### Secondary outcomes

Only one study reported cardiovascular-specific mortality with respect to blood pressure and frailty [17]. In this study, lower diastolic blood pressure was associated with increased cardiovascular disease mortality in patients over the age of 80 years or in those with slower walking speed. By contrast, high diastolic blood pressure was reported to be associated with higher cardiovascular disease mortality in patients under the age of 72 years, and in those without physical and cognitive impairment. Data were not available for the other pre-specified secondary outcomes.

### Sensitivity analyses

A high or uncertain risk of bias was identified in four studies in the measurement of exposure. The exclusion of these studies did not change the pooled estimates significantly in any of the four meta-analyses. The exclusion from meta-analyses of the largest study (*n* = 5,375) [23] for categorical comparisons of diastolic BP, changed the pooled estimate for those with frailty (HR 0.84 95% CI 0.70 to 1.02) and for those without frailty (HR 1.08 95% CI 0.7 to 1.68). However, there was no significant change in pooled estimates of categorical comparisons of systolic BP, continuous systolic or diastolic BP comparisons with and without frailty.

### Effect modification

Six studies assessed whether frailty had an interaction with the association of blood pressure and mortality. Three reported a significant interaction with systolic blood pressure (*p* < 0.05 [22–24]), three reported no significant difference [17, 20, 21]. Three studies assessed whether anti-hypertensive treatment [16, 18, 24] or self-reported diagnosis of hypertension [24] modified the effect of frailty on blood pressure and mortality, but found no evidence of a significant interaction. One study stratified continuous comparisons of systolic BP by antihypertensive treatment and found that frailty did not modify the effect [20]. Five studies reported sensitivity analyses to exclude those dying within 1 year [16, 18, 20, 22] and 2 years [23], to test for evidence of reverse causality, all showing no effect on the summary estimates.

## Discussion

In this meta-analysis of 21,906 participants across nine cohort studies, we found that for older people with frailty, a systolic blood pressure less than 140 mm Hg was not associated with a difference in mortality compared to a systolic blood pressure greater than 140 mm Hg. In contrast, in older people without frailty, a systolic blood pressure less than 140 mm Hg was associated with a 14% lower risk of death compared to a systolic blood pressure more than 140 mm Hg.

There was no association between diastolic blood pressure and mortality in older people overall (*n* = 10,267), and this did not change when accounting for frailty. When measuring blood pressure as a linear variable, there was no difference in association with higher systolic (*n* = 12,280) or diastolic blood pressure (*n* = 11,668) and mortality after adjustment.

### Strengths and weaknesses

Our robust, inclusive search strategy identified studies that recruited an average of 58% of eligible participants. The study populations were larger and more representative of community-dwelling older people than recent randomised control trials [4, 26]. We compared neighbouring categories at thresholds defined by current NICE guidelines [15]. Our synthesis of adjusted data provides greater confidence in the meta-analysis findings.

Whilst we set out to investigate a number of other outcomes in addition to mortality the available studies did not report non-fatal outcomes to enable pooled estimates of risk to be calculated. The proportion of the study population who were care home residents was reported in a minority of included studies, limiting conclusions about this important group with advanced frailty.

There was variation in the criteria for diagnosis of hypertension and the thresholds for treatment. All studies measured blood pressure at one sitting, but measurement error and short-term variability of blood pressure mean that single readings are unreliable. Whilst there was no evidence of a linear dose effect of blood pressure, we could not exclude a nonlinear association, due to a lack of reported data, which could be relevant considering the reported J-shaped relationship between blood pressure and outcomes in other populations [27].

Throughout the meta-analyses, we dichotomised patients as either frail or non-frail to allow us to compare patients across a number of different frailty measures, however there is much evidence that frailty is graded. Frailty was inconsistently defined across studies with the use of a variety of measures.

It is possible that the association reported in this review may be the result of reverse causality, i.e. that low blood pressure in the context of frailty may be a marker of proximity to death due to failure of multiple physiological systems [28]. Although several studies performed sensitivity analyses to test this, the numbers included were small, and therefore the analysis to determine this may have been underpowered.

### Findings in context of wider research literature

Two RCTs have included older people and measured frailty [4, 26]. Consistent with the findings from our meta-analysis, these two trials have reported persistent benefit from low blood pressure extending into old age for those without frailty. In contrast to findings from our meta-analysis, the trials have reported no evidence that frailty modifies the beneficial effects of BP treatment on mortality reduction [4, 26]. However, there are concerns regarding the generalisability of trial findings because of their narrow eligibility criteria [29]. For example, older people with impaired activities of daily living and care home residents were excluded, limiting generalisability across the spectrum of frailty. Furthermore, both RCTs reported retrospective secondary analyses of the original trial data that were not pre-specified or statistically powered for the analyses by frailty status.

### Implications

Future observational research studies should investigate the association between blood pressure and outcomes of importance to older people living with frailty, including independence, falls, and quality of life. A definitive trial is required to resolve whether treatment is effective in this population across the frailty spectrum, including those with advanced frailty who are dependent in instrumental and basic activities of daily living.

## Conclusions

This systematic review of observational studies has identified an association between low systolic blood pressure and lower all-cause mortality in older adults without frailty, but not in those with frailty. Our findings indicate that in the absence of frailty, blood pressure targets should be considered independently of age. In the presence of frailty there is ongoing uncertainty, but available evidence indicates a personalised approach based on individual circumstances is appropriate.

## Supplementary Material

Appendix 1

Appendix 2

Appendix 3

## Figures and Tables

**Table 1:** Characteristics of included studies

Study	Study size	Exclusion criteria	Frailty measure & threshold	Blood pressure (mm Hg)				Confounders in addition to Age & Sex	
	Mean duration								
				Readings	Categorical measures		Continuous measures		
	Recruitment				Systolic	Diastolic			
**Gutierrez-Misis** [16]	649 6 yrs 39%	Unable to consent; moved away; early death	Gait 0.8 m/s	2	<120 ≥120 & <140 ≥140	<80 ≥80 & <90 ≥90		BMI; cholesterol; depression; CCF; cognition; stroke	
**Hospers** [17]	1,411 11 yrs 53%	Refusal; early death; ‘too frail’; not contactable	Gait 0.8 m/s	1	≤120v >120 & ≤140 >140	< 70 ≥70 & <90 ≥90		Education; BMI; smoking; alcohol consumption; cholesterol; cardiovascular disease; diabetes; and antihypertensive drug use	
**Lv** [24]	4,658 3 yrs 60%	Age <79 y; missing data; early death	OFI >2/3	2	≤ 107 ≤107 vs >154 >154	<70 ≥70 & ≥90 >90		Marital status; education; residence; income; smoking; alcohol; cognitive impairment; restrictions on ADL; poor vision; BMI; central obesity; DM; CVD; stroke; respiratory disease; cancer.	
**Odden** [18]	2,340 6 yrs 37%	Institutional living; early death	Gait 0.8 m/s	3–4	< 140 vs. ≥140	< 90 vs. ≥90	10 mmHg difference	CCF; CHD; cholesterol; education; race; smoking; stroke; sur vey year	
**Peralta** [19]	3,547 8 yrs 20%	Unable to consent; moved away; cancer	Gait 0.8 m/s	3	<120 ≥120 & <150 ≥150	<65 ≥65 & <80 ≥80	10 mmHg difference	Education; race; smoking physical activity; BMI; cholesterol; cystatin C; hypertension medications; and sBP or dBP respectively	
**Streit** [20]	570 5 yrs 81%	Early death; missing data	Grip *not defined*	2			10 mmHg difference	CVD; BP medications	
**Vaes** [21]	541 5 yrs 92%*	Dementia; palliative care; emergency	GFI 6+/15 Fried 3+/5 Puts 3+/9	2			10 mmHg difference	Education	
**Weidung** [22]	745 3 yrs 58%	Early death; missing data	Gait 0.5 m/s	1	≤ 125 >125 & <140 ≥140 & <150 ≥150 & <165 ≥165	<70 ≥70 & ≤ 75 ≥75 & <80 ≥80		Follow-up time; CCF; AF; MI; cancer; depression; angina; BMI; MMSE score; adjusted for care facility residency; living alone; education; CVD; hip fracture; specific drugs and number of drugs.	
**Wu** [23]	7,492 6 yrs 79%	Institutional living; missing data; fast gait	Gait *f* 0.52 *m* 0.6 m/s; Grip *f* 16 kg *m* 26 kg	3	<140 vs. ≥140 <150 vs. ≥150	< 90 vs. >90	10 mmHg difference	BMI; BP medication; cancer; cardiac disease; HbA1c; CRP; cystatin C; diabetes; education; ethnicity; smoking; stroke	

Characteristics include study population, frailty, blood pressure and confounder variables. ADL = activities of daily living; AF = atrial fibrillation; BP = blood pressure; BMI = body mass index; CCF = congestive cardiac failure; CHD = coronary heart disease; CRP = C-reactive protein; CVD = cerebrovascular disease; DM = diabetes mellitus; dBP = diastolic blood pressure; f = female; Gait = Gait speed; Grip = Grip strength; GFI = Groeningen Frailty Index; HbA1c = glycated haemoglobin; LAPAQ = Longitudinal Aging Study Amsterdam (LASA) Physical Activity Questionnaire; MI = myocardial infarction; MMSE = Mini-mental status exam; m = male; n = sample size; OFI = osteoporotic fracture index; sBP = systolic blood pressure; yrs = years.

* Estimate using information presented, but exact figures not provided.

**Table 2:** Risk of bias assessment

Study	Selection of participants	Confounding variables	Measurement of exposure	Blinding of outcome	Incomplete outcome data	Selective outcome reporting	Overall judgement
Gutierrez-Misis [16]	Low	Low	High	Low	Low	Unclear	Mod
Hospers [17]	Unclear	Low	Low	Low	Low	Unclear	Low
Lv [24]	Low	Unclear	Low	Low	Low	Unclear	Low
Odden [18]	Low	Low	Low	Low	Low	Unclear	Low
Peralta [19]	Low	Low	Unclear	Low	Low	Unclear	Low
Streit [20]	Low	Low	High	Low	Low	Unclear	Mod
Vaes [21]	Low	Low	Low	Low	Low	Unclear	Low
Weidung [22]	Low	Low	High	Low	Low	Unclear	Mod
Wu [23]	Unclear	Unclear	Low	Low	Low	Unclear	Low

Risk of bias assessment using the RoBANS tool [13].

## Key points

- This systematic review and meta-analysis categorised and synthesised blood pressure measurements according to NICE guidelines.
- Results indicate that mortality is lower for non-frail older people when systolic blood pressure is less than 140 mm Hg.
- Results indicate no mortality difference for older people with frailty when systolic blood pressure is less than 140 mm Hg.
- There is a lack of evidence of the association between blood pressure and non-fatal outcomes in older people with frailty.
- Identification of frailty is a potentially useful way to inform shared decision making for blood pressure treatment in older age.
