# Supplementary material for: Is the association between blood pressure and mortality in older adults different with frailty? A systematic review and meta-analysis
Source: Age Ageing. Author manuscript; Available in PMC 2025 Oct 14. (PMC7618252; doi:10.1093/ageing/afz072)
Supplement: Appendix 1 [file EMS209394-supplement-Appendix_1.docx]

# Appendix 1: MOOSE Guideline (1)

| **Item No** | **Recommendation** | **Reported on Page No** |
| --- | --- | --- |
| Reporting of background should include | | |
| 1 | Problem definition | 3 |
| 2 | Hypothesis statement | 3/4 |
| 3 | Description of study outcome(s) | 5 |
| 4 | Type of exposure or intervention used | 5 |
| 5 | Type of study designs used | 5 |
| 6 | Study population | 5 |
| Reporting of search strategy should include | | |
| 7 | Qualifications of searchers (eg, librarians and investigators) | 6 |
| 8 | Search strategy, including time period included in the synthesis and key words | 6, Appendix 2 |
| 9 | Effort to include all available studies, including contact with authors | 6 |
| 10 | Databases and registries searched | 6 |
| 11 | Search software used, name and version, including special features used (eg, explosion) | 6 |
| 12 | Use of hand searching (eg, reference lists of obtained articles) | 6 |
| 13 | List of citations located and those excluded, including justification | Figure 1 |
| 14 | Method of addressing articles published in languages other than English | 6 |
| 15 | Method of handling abstracts and unpublished studies | 6 |
| 16 | Description of any contact with authors | 6 |
| Reporting of methods should include | | |
| 17 | Description of relevance or appropriateness of studies assembled for assessing the hypothesis to be tested | 6 |
| 18 | Rationale for the selection and coding of data (eg, sound clinical principles or convenience) | 6 |
| 19 | Documentation of how data were classified and coded (eg, multiple raters, blinding and interrater reliability) | 6 |
| 20 | Assessment of confounding (eg, comparability of cases and controls in studies where appropriate) | 6 |
| 21 | Assessment of study quality, including blinding of quality assessors, stratification or regression on possible predictors of study results | 6 |
| 22 | Assessment of heterogeneity | 7 |
| 23 | Description of statistical methods (eg, complete description of fixed or random effects models, justification of whether the chosen models account for predictors of study results, dose-response models, or cumulative meta-analysis) in sufficient detail to be replicated | 7 |
| 24 | Provision of appropriate tables and graphics | Figure 2, Tables I, II |
| Reporting of results should include | | |
| 25 | Graphic summarizing individual study estimates and overall estimate | Figure 2 |
| 26 | Table giving descriptive information for each study included | Table I |
| 27 | Results of sensitivity testing (eg, subgroup analysis) | 12 |
| 28 | Indication of statistical uncertainty of findings | 10-11 |
| 29 | Quantitative assessment of bias (eg, publication bias) | 7 |
| 30 | Justification for exclusion (eg, exclusion of non-English language citations) | 8 |
| 31 | Assessment of quality of included studies | 9-10 |
| Reporting of conclusions should include | | |
| 32 | Consideration of alternative explanations for observed results | 15 |
| 33 | Generalization of the conclusions (ie, appropriate for the data presented and within the domain of the literature review) | 14,16 |
| 34 | Guidelines for future research | 16 |
| 35 | Disclosure of funding source | online |
|  | | |

**Appendix 2: Search Strategy**

Database: Ovid MEDLINE(R) Epub Ahead of Print, In-Process & Other Non-Indexed Citations, Ovid MEDLINE(R) Daily and Ovid MEDLINE(R) <1946 to Present>

Search Strategy:

--------------------------------------------------------------------------------

1 late* life.tw. (16019)

2 age factors/ (452959)

3 (frail* or sarcop?eni* or prefrailty).mp. (26267)

4 Sarcopenia/ (2686)

5 function* status.tw. (24184)

6 activities of daily living.tw. (23276)

7 "activities of daily living"/ (63462)

8 (physical adj3 function).tw. (14334)

9 Hypertension/ (238653)

10 ((high or elevat*or rais*) adj2 blood pressure).tw. (16209)

11 (blood pressure adj6 goal?).mp. (2028)

12 Blood Pressure Determination/ (27584)

13 epidemiologic studies/ (8301)

14 exp case control studies/ (998349)

15 exp cohort studies/ (1905682)

16 case control.tw. (116518)

17 (cohort adj (study or studies)).tw. (164282)

18 cohort analy*.tw. (6584)

19 (follow up adj (study or studies)).tw. (48680)

20 (observational adj (study or studies)).tw. (85828)

21 Longitudinal.tw. (219409)

22 retrospective.tw. (451275)

23 cross sectional.tw. (291356)

24 cross-sectional studies/ (283885)

25 survey.tw. (466607)

26 survey/ (429354)

27 or/13-26 [epidemiology filter] (3317120)

28 or/9-12 (264635)

29 or/1-8 (587455)

30 27 and 28 and 29 (5745)

**Appendix 3: Method of Extraction for Meta-Analyses**

Comparison to a standard reference

Where we have two hazard ratios comparing groups B and C to group A, and we want a hazard ratio comparing group C to group B:

|  | A | B | C |
| --- | --- | --- | --- |
| HR (95% CI) | 1 | 0.89 (0.62, 1.28) | 0.94(0.65,1.35) |

We will find HRs and SEs, then find SE for log difference, C − B

First we switch A & B:

log(0.89) = −0.11653382

log(0.62) = −0.4780358

log(1.28) = 0.24686008

Switching the signs of these gives the ratio for the log HR for A with B as standard.

Now find the standard error:

(log(1.28) − log(0.62)) /(2*1.96) = 0.18492242

Note that “*” means “multiply”. Later, “^2” means “raised to the power 2” or “squared” and “sqrt” means “square root”.

Convert back to natural scale and find the confidence interval:

exp(−log(0.89) − 1.96*(log(1.28) − log(0.62)) /(2*1.96)) = 0.78198938

exp(−log(0.89) + 1.96*(log(1.28) − log(0.62)) /(2*1.96)) = 1.6144297

Hence the estimate is 1.12 and the 95% confidence interval is 0.78 to 1.61.

Now for C with B as standard, which is more difficult. The problem is that we need to combine both HRs.

log(0.94) = −0.0618754

log(0.65) = −0.43078292

log(1.35) = 0.30010459

SE: (log(1.35) − log(0.65))/(2*1.96) = 0.1864509

Difference, C – B:

log(HR) = log(0.94) − log(0.89) = 0.05465841

HR = exp(log(0.94) − log(0.89)) = 1.0561798

Now, we can calculate the SE for the difference, by taking the square root of the sum of the squares of the two SEs. However, there is an assumption, that the estimates for B/A and C/A are independent, which is clearly false.

SE(difference) = sqrt( ((log(1.35) − log(0.65))/(2*1.96))^2 + ((log(1.28) − log(0.62)) /(2*1.96))^2) = 0.26260281

Transform back and get 95% CI:

exp( log(0.94) − log(0.89) − 1.96*sqrt( ((log(1.35) − log(0.65))/(2*1.96))^2 + ((log(1.28) − log(0.62)) /(2*1.96))^2)) = 0.63125644

exp( log(0.94) − log(0.89) + 1.96*sqrt( ((log(1.35) − log(0.65))/(2*1.96))^2 + ((log(1.28) − log(0.62)) /(2*1.96))^2)) = 1.7671356

The estimated 95% CI for the HR is 0.631 to 1.767.

This is plausible, in that it contains the estimate 1.056 comfortably. It looks wide, compare to the Cis for B/A and C/A. This is because of the false assumption of independence. If we had all the data, we could allow for the dependence and obtain a smaller SE and narrower confidence interval. However, we don’t. So we would use this an approximation, with the caveat that the standard error may be too big, which may slightly reduce the contribution of this study to the overall estimate.

**References**

1. Stroup, D.F. et al. Meta-analysis of observational studies in epidemiology: a proposal for reporting. Meta-analysis Of Observational Studies in Epidemiology (MOOSE) group. *JAMA.* 2000, **283**(15), pp.2008-12.
