# Supplementary material for: Is the association between blood pressure and mortality in older adults different with frailty? A systematic review and meta-analysis
Source: Age Ageing. Author manuscript; Available in PMC 2025 Oct 14. (PMC7618252; doi:10.1093/ageing/afz072)
Supplement: Appendix 2 [file EMS209394-supplement-Appendix_2.docx]

**Appendix 2: Search Strategy**

Database: Ovid MEDLINE(R) Epub Ahead of Print, In-Process & Other Non-Indexed Citations, Ovid MEDLINE(R) Daily and Ovid MEDLINE(R) <1946 to Present>

Search Strategy:

--------------------------------------------------------------------------------

1 late* life.tw. (16019)

2 age factors/ (452959)

3 (frail* or sarcop?eni* or prefrailty).mp. (26267)

4 Sarcopenia/ (2686)

5 function* status.tw. (24184)

6 activities of daily living.tw. (23276)

7 "activities of daily living"/ (63462)

8 (physical adj3 function).tw. (14334)

9 Hypertension/ (238653)

10 ((high or elevat*or rais*) adj2 blood pressure).tw. (16209)

11 (blood pressure adj6 goal?).mp. (2028)

12 Blood Pressure Determination/ (27584)

13 epidemiologic studies/ (8301)

14 exp case control studies/ (998349)

15 exp cohort studies/ (1905682)

16 case control.tw. (116518)

17 (cohort adj (study or studies)).tw. (164282)

18 cohort analy*.tw. (6584)

19 (follow up adj (study or studies)).tw. (48680)

20 (observational adj (study or studies)).tw. (85828)

21 Longitudinal.tw. (219409)

22 retrospective.tw. (451275)

23 cross sectional.tw. (291356)

24 cross-sectional studies/ (283885)

25 survey.tw. (466607)

26 survey/ (429354)

27 or/13-26 [epidemiology filter] (3317120)

28 or/9-12 (264635)

29 or/1-8 (587455)

30 27 and 28 and 29 (5745)
