# Supplementary material for: Is the association between blood pressure and mortality in older adults different with frailty? A systematic review and meta-analysis
Source: Age Ageing. Author manuscript; Available in PMC 2025 Oct 14. (PMC7618252; doi:10.1093/ageing/afz072)
Supplement: Appendix 3 [file EMS209394-supplement-Appendix_3.docx]

**Appendix 3: Method of Extraction for Meta-Analyses**

Comparison to a standard reference

Where we have two hazard ratios comparing groups B and C to group A, and we want a hazard ratio comparing group C to group B:

|  | A | B | C |
| --- | --- | --- | --- |
| HR (95% CI) | 1 | 0.89 (0.62, 1.28) | 0.94(0.65,1.35) |

We will find HRs and SEs, then find SE for log difference, C − B

First we switch A & B:

log(0.89) = −0.11653382

log(0.62) = −0.4780358

log(1.28) = 0.24686008

Switching the signs of these gives the ratio for the log HR for A with B as standard.

Now find the standard error:

(log(1.28) − log(0.62)) /(2*1.96) = 0.18492242

Note that “*” means “multiply”. Later, “^2” means “raised to the power 2” or “squared” and “sqrt” means “square root”.

Convert back to natural scale and find the confidence interval:

exp(−log(0.89) − 1.96*(log(1.28) − log(0.62)) /(2*1.96)) = 0.78198938

exp(−log(0.89) + 1.96*(log(1.28) − log(0.62)) /(2*1.96)) = 1.6144297

Hence the estimate is 1.12 and the 95% confidence interval is 0.78 to 1.61.

Now for C with B as standard, which is more difficult. The problem is that we need to combine both HRs.

log(0.94) = −0.0618754

log(0.65) = −0.43078292

log(1.35) = 0.30010459

SE: (log(1.35) − log(0.65))/(2*1.96) = 0.1864509

Difference, C – B:

log(HR) = log(0.94) − log(0.89) = 0.05465841

HR = exp(log(0.94) − log(0.89)) = 1.0561798

Now, we can calculate the SE for the difference, by taking the square root of the sum of the squares of the two SEs. However, there is an assumption, that the estimates for B/A and C/A are independent, which is clearly false.

SE(difference) = sqrt( ((log(1.35) − log(0.65))/(2*1.96))^2 + ((log(1.28) − log(0.62)) /(2*1.96))^2) = 0.26260281

Transform back and get 95% CI:

exp( log(0.94) − log(0.89) − 1.96*sqrt( ((log(1.35) − log(0.65))/(2*1.96))^2 + ((log(1.28) − log(0.62)) /(2*1.96))^2)) = 0.63125644

exp( log(0.94) − log(0.89) + 1.96*sqrt( ((log(1.35) − log(0.65))/(2*1.96))^2 + ((log(1.28) − log(0.62)) /(2*1.96))^2)) = 1.7671356

The estimated 95% CI for the HR is 0.631 to 1.767.

This is plausible, in that it contains the estimate 1.056 comfortably. It looks wide, compare to the Cis for B/A and C/A. This is because of the false assumption of independence. If we had all the data, we could allow for the dependence and obtain a smaller SE and narrower confidence interval. However, we don’t. So we would use this an approximation, with the caveat that the standard error may be too big, which may slightly reduce the contribution of this study to the overall estimate.

**References**

1. Stroup, D.F. et al. Meta-analysis of observational studies in epidemiology: a proposal for reporting. Meta-analysis Of Observational Studies in Epidemiology (MOOSE) group. *JAMA.* 2000, **283**(15), pp.2008-12.
